# Small heterodimer partner 1 directly interacts with NS5A viral protein and has a key role in HCV related liver cell transformation

**DOI:** 10.18632/oncotarget.12144

**Published:** 2016-09-20

**Authors:** Beatrice Conti, Cristiana Porcu, Carmela Viscomi, Antonella Minutolo, Susan Costantini, Marco Corazzari, Gino Iannucci, Barbara Barbaro, Clara Balsano

**Affiliations:** ^1^ Laboratory of Molecular Virology and Oncoloy, Francesco Balsano Foundation, ex A. Cesalpino Foundation, Rome, Italy; ^2^ Institute of Biology and Molecular Pathology (IBPM) – CNR (National Research Council), Rome, Italy; ^3^ Department of Biology, University of Rome ‘Tor Vergata’, Rome, Italy; ^4^ CROM, Istituto Nazionale Tumori “Fondazione G.Pascale”, IRCSS, Napoli, Italy

**Keywords:** HCV, liver steatosis, SHP1, NS5A, HCC

## Abstract

HCV life cycle is strictly correlated with the hepatocyte lipid metabolism; moreover, the progression of HCV chronic hepatitis is accelerated by the presence of liver steatosis. Among the steatogenic genes deregulated during the HCV infection one of the most attractive is the Small Heterodimer Protein 1 (SHP1; NR0B2), that is involved in a remarkable number of metabolic functions. HCV NS5A is an essential and integral component of the HCV membranous-web replicon complex (RC) and plays an essential role to transfer the viral genome from the RCs to the surface of the lipid droplets (LDs) that, in turn, play a key function during HCV life cycle.

With the help of a HCV infection model, we demonstrate a functional interaction between SHP1 and HCV NS5A protein. SHP1 silencing (siSHP1) reversed the pro-oncogenic effects of HCV infection, inducing a significant decrease in liver lipid accumulation and in NS5A protein expression. Moreover, siSHP1 causes a strong modulation of some genes involved in HCV-related EMT, such as: HNF4, a central regulators of hepatocyte differentiation, E-Cadherin, SNAILs.

Our data suggest that SHP1 results not only to be strictly connected to the pathogenesis of HCV-related liver steatosis, but also to its progression towards the liver transformation.

## INTRODUCTION

The hepatitis C virus (HCV) infection is a major cause of chronic liver disease worldwide [1]. HCV is an enveloped, single-stranded, positive-sense RNA virus, belonging to the Hepacivirus genus of the Flaviviridae family. The viral RNA encodes a large polyprotein precursor of approximately 3100 amino acids that is co- and/or post- translationally cleaved by both host and viral proteases into three mature structural proteins, building the capsid, (core, E1, E2), and seven non-structural (NS) proteins involved in virus replication (p7, NS2, NS3, NS4A, NS4B, NS5A and NS5B). Viral genome replication occurs in replication complexes (RCs) localized on the cytoplasmic face of specialized membranes of the endoplasmic reticulum, the so called: membranous-web [2].

An interesting aspect of the HCV life cycle is its strict correlation with the hepatocyte lipid metabolism. Left untreated, chronic hepatitis C can progress towards cirrhosis and hepatocellular carcinoma (HCC) and this process is accelerated by the presence of liver steatosis. Host lipid metabolism, with lipoproteins, lipid droplets and host cofactors, favors HCV replication, morphogenesis, and secretion [3, 4]. Accordingly, HCV replication, virion assembly and release, perturb lipid metabolism of infected hepatocytes [5, 6]. In particular, NS5A viral protein plays an essential role in transferring the viral genome from the RCs to the surface of the lipid droplets [7].

Lipid homeostasis is tightly controlled by a complex network of transcriptional programs [8, 9]. Among them, Liver X Receptor-dependent intracellular pathways modulates genes involved in reverse cholesterol transport and hepatic cholesterol metabolism, such as Peroxisome Proliferator-Activated Receptor gamma (PPAR-γ) and Liver X Receptor alpha (LXRα), increasing the synthesis of fatty acids by the up-regulation of Sterol regulatory element-binding proteins 1c (SREBP-1c) and Fatty acid synthase (FASN) [10–11].

A remarkable number of the above described metabolic pathways are regulated by the Small heterodimer partner 1 (SHP1; NR0B2), predominantly expressed in liver and gallbladder. SHP1 is an orphan member of the nuclear receptor superfamily, which targets, as a transcriptional co-repressor, other nuclear receptors (NRs) [12]. The discovery of SHP1 dates back to 1996; since then, this orphan NR has been identified as a key transcriptional regulator of signaling pathways involved in fundamental biological functions and metabolic processes [13]. SHP1 has a pleiotropic role in the pathology of chronic liver disease and the alteration of its functions has been coupled not only with cholestasis, diabetes and obesity but also with cancer [14].

SHP1 executes its regulatory function through protein–protein interactions with NRs and transcription factors (TFs), inhibiting their transcriptional activities through co-activator competition followed by co-repressor recruitment [15, 16]. Many Nuclear Receptors (NRs), bind and specifically activate molecules through ligand-binding domains (LBDs), interacting, directly or indirectly, with this co-repressor to mediate transcriptional regulation of lipid metabolism [17].

Finally, although SHP1 has been described to play antitumor role in the development of cancer [18] the heterogeneity of HCC subtypes linked to different oncogenic pathways, still represent the major barriers for HCC study.

Our study is focused on understanding the involvement of SHP1 in the progression of hepatitis C toward HCC.

## RESULTS

### SHP1 expression in HCV infected patients

Our previously published data indicated that HCV infected patients were characterized by a deep deregulation of some genes involved in the control of lipid metabolism. Accordingly, we demonstrated that this feature was restricted to Hepatitis C virus infection, whereas HBV patients had different features [19]. Among all, the previously analyzed steatogenic genes (i.e. LXRα, SREBP1c, FASN), we sought to better investigate the role of SHP1 during HCV infection.

**Figure 1:**
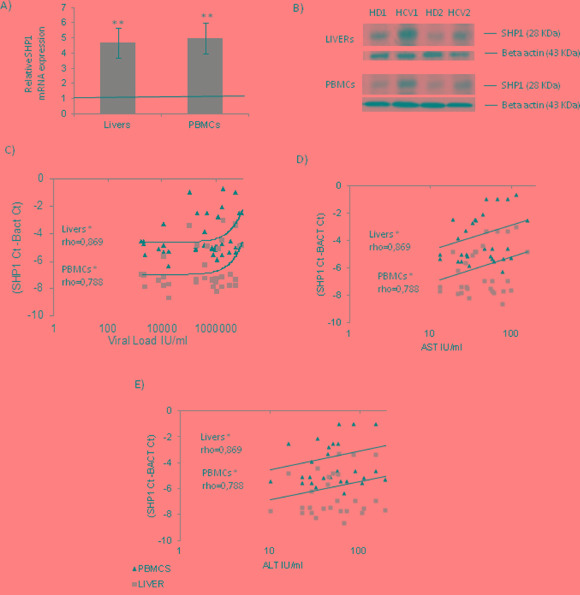
SHP1 expression in chronic HCV patients **A.** mRNA expression levels of SHP1 in livers and PBMCs from HCV infected patients (the results are represented as mean values ± S.D, **p<0.01). **B.** Western blot analysis of SHP1 in livers and PBMCs of HCV infected patients. The presented results are obtained by comparing healthy donors (HDs) with HCV infected patients with different viral loads (VLs). Represented experiments are two examples of what happen in the liver and PBMCs of the studied subjects. HD1: Healthy Donor 1, HCV 1: HCV Infected patient 1, HD2: Healthy Donor 2 and HCV2: HCV infected patient 2. **C**, **D**, **E.** Correlation between SHP1 expression in HCV+ livers/PBMCs and plasma ALT, AST levels and Viral Loads (VL), respectively. The Spearman's rho was evaluated with a Bonferroni's correction of 0.000568, (livers: rho values 0.869, *p<0.05; PBMCs: rho values 0.788, *p<0.05).

First, we studied the expression of SHP1 in liver biopsies and PBMCs isolated from 40 HCV infected patients, highlighting that both showed its significant (** p<0.01) up-regulation (Figure 1A and 1B, fold change HDs=1, HCV livers 4.56± 3.2; PBMCs 5.16± 3.45).

SHP1 mRNA expression in HCV patients positively correlates with the plasma levels of ALT and AST (*p<0.05), as well as with HCV RNA (VL) (Figure 1C, 1D and 1E), suggesting a possible key role of SHP1 in HCV-mediated pathogenesis.

### Relationship between SHP1 and NS5A in *in vitro* model of HCV infection

**Figure 2:**
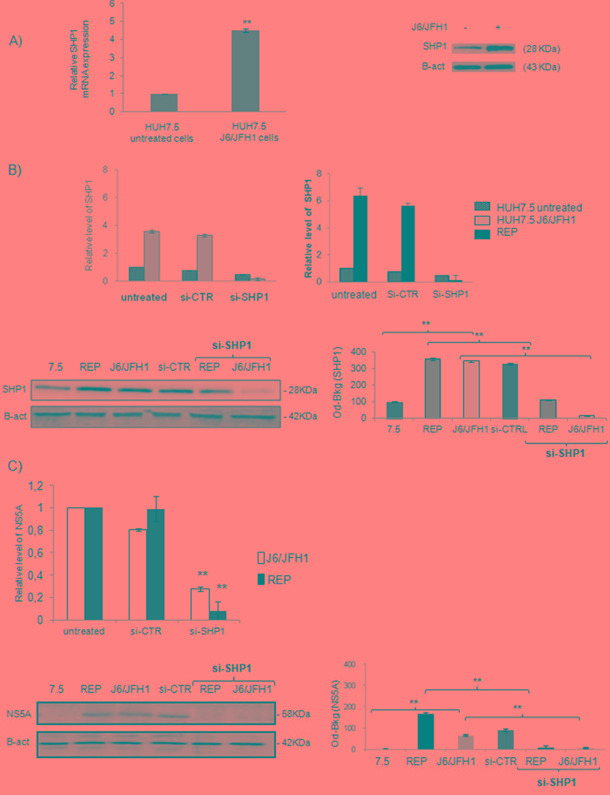
Relationship between SHP1 and NS5A in *in vitro* model of HCV infection **A.** SHP1 expression levels in Huh7.5 J6/JFH1 HCV infected cells. mRNA expression level and WB analyses of SHP1, 72 hrs post-infection. The results derived from 5 independent experiments. Data are expressed as mean ± S.D, **p<0.01. **B.** SHP1 interfering efficacy in both Huh7.5 J6/JFH1 and JFH1 A4 replicon system, after 72 hours of infection: mRNA expression levels, WB and relative densitomentric analysis of SHP1 after its silencing. Data are expressed as mean values ± S.D, **p<0.01. Beta-actin is used as a loading control. Immunoblot is representative of five independent experiments. **C.** mRNA expression levels, WB assay and relative densitomentric analysis of NS5A in Huh7.5 J6/JFH1 cells and JFH1 A4 replicon system, in presence or not of siSHP1. Beta-actin is used as a loading control. Immunoblot is representative of five independent experiments (**p<0.01).

To confirm the results obtained “*ex vivo*”, we established the in *in vitro* model of HCV infection: Huh7.5 cells were infected with the chimera J6/JFH1. 72 hrs post-infection we observed an up-regulation of HCV IRES and the expression of the HCV Core and NS5A proteins (data not shown). SHP1 was up-regulated in the HCV infected cells as reported in Figure 2A and 2B (** p<0.01). We confirmed, the up-regulation of SHP1 also in JFH1 A4 replicon system, expressing all the HCV non-structural proteins (data not shown). SHP1 up-regulation was specific to HCV infection, since was not found in non-HCV infection model (Supplemental Material, Figure S1).

To investigate the role of SHP1 during HCV infection and in HCV-related pathogenesis, we modulated SHP1 transcription using specific RNAs interference (siRNA-SHP1). We tested 4 different SHP1 siRNA, deciding to use A4 SHP1 siRNA that was able to significantly reduce the SHP1 transcript and protein, and didn’t affect cell viability (Supplemental Material, Figure S2). In Figure 2B we showed that mRNA and protein levels of SHP1 were reduced by si-SHP1 in both HuH7.5 JFH1/J6 infected cells and in the JFH1 A4 replicon system. It was interesting to note that, following the SHP1 silencing, NS5A mRNA levels were significantly reduced both in HuH7.5 JFH1/J6 infected cells (** p<0.01) and in the JFH1 A4 replicon system (Figure 2C). Among all the viral proteins, only NS5A was significantly reduced after siSHP1 interference (data not shown).

### Direct interaction between SHP1 and NS5A HCV viral protein

**Figure 3:**
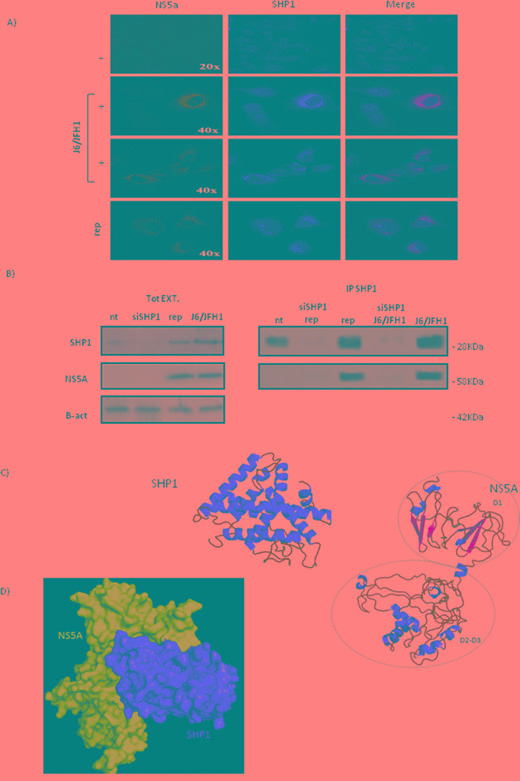
Interaction between SHP1 and NS5A **A.** Confocal double immunofluorescence microscopy of: NS5A and endogenous SHP1 on Huh7.5 (first horizontal panel), Huh7.5 infected J6/JFH1 infected cells (second and third panel), and replicon system (fourth horizontal panel), 72 hours post infection. Merge of the two analyzed proteins is on the right. **B.** SHP1 and NS5A coimmuno-precipitation assay, in Huh7.5 J6/JFH1 cells and JFH1 A4 replicon systems. On the left, WB of total protein extracts; on the right, immuno-precipitation in Huh7.5 J6/JFH1 cells and JFH1 A4 replicon systems before and after si-SHP1. Data are representative of three independent experiments. **C.** Model of SHP1 and NS5A: alpha-helices and beta-sheets are depicted by red and yellow, respectively. **D.** Model of the complex between SHP1 (in red) and NS5A (in cyan) by surface representation.

To verify if the relationship between SHP1 and NS5A protein expression was due to a direct or indirect link, we looked at the intracellular localization of SHP1 and NS5A, in HuH7.5 JFH1/J6 infected cells and replicon system, by confocal immunofluorescence microscopy (Figure 3A). The infected cells displayed a delocalization of SHP1 protein (red) from the nucleus to the perinuclear zone. Moreover, we highlighted a co-localization between endogenous SHP1 and NS5A (green) HCV non-structural protein. We performed a co-immunoprecipitation assay to deepen the direct interaction between SHP1 and NS5A HCV protein (Figure 3B). The immunoprecipitation of SHP1 and NS5A strengthen the possibility of a protein-protein interaction between SHP1 and NS5A viral protein.

### Computational model of interaction between SHP1 and NS5A HCV viral protein

To define the domains involved in the interaction between SHP1 and NS5A, we performed a computational analysis to predict the precise domains of interaction. Firstly, we made a modeling of the single proteins, and then we searched for the complex.

The three-dimensional model of SHP1 was built according to an approach combining fold recognition and comparative modeling as reported in the Methods section. On the basis of six templates found by Phyre server (Supplemental Material, Figure S3), the final model of SHP1 was obtained. It had a percentage of residues in the most favored regions of the Ramachandran Plot equal to 92.2% and a ProSA Z-score of -4.59. As visible in Figure 3C, SHP1 shows a alpha-structure characterized by nine alpha-helices.

A recent publication regard NMR investigation on the NS5A C-terminal residues 191–447 (NS5A-D2D3) has evidenced the presence of local residual (alpha-helical and beta-turn) structure. In details, there were evidenced four peptide regions (195–220, 251–266, 292–306 and 370–378) showing the characteristic structured α-helices signature [20]. On the basis of experimental data, we modeled the 3D-structure of the D2D3 domains by the I-TASSER server via *ab initio* modeling [21], and by imposing the location of the helix regions found in NMR experiments. We selected the best model on the basis of two criteria: i) the lower number of gaps and ii) the higher number of residues in helix in similar positions in respect with NMR data (Supplemental Material, Table S1). The model 3 was chosen and used to create the complete structure of NS5A together with the X-Ray structure of D1 domain (PDB:3FQM). The final 3D-structure of C-terminal portion of NS5A showed 87.8 % of residues in the favored regions of the Ramachandran Plot and an energetic Z-score of -4.37 (Figure 3C).

To verify the surface complementarity between SHP1 and NS5A and to find the possible interaction regions between the two proteins, we performed a molecular docking by PathDoch program. We selected the best pose on the basis of the higher energetic score and, hence, of higher number of Hydrogen bonds and interaction residues (Figure 3D). From the analysis of the different complexes we selected the best complex that had at the interface 21 residues of NS5A and 29 residues of NRP-1, also stabilized by three Hydrogen bonds: Ser41(NS5A) and Gly248(SHP1), Ile44(NS5A) and Arg71 (SHP1) and His55 (NS5A) and Arg36 (SHP1). Our computational data indicate thet the interaction between SHP1 and NS5A occurs mainly between SHP1 and the D1 domain of NS5A, giving the opportunity to counteract the noxious interaction.

### SHP1 silencing affects HCV related lipid accumulation

**Figure 4:**
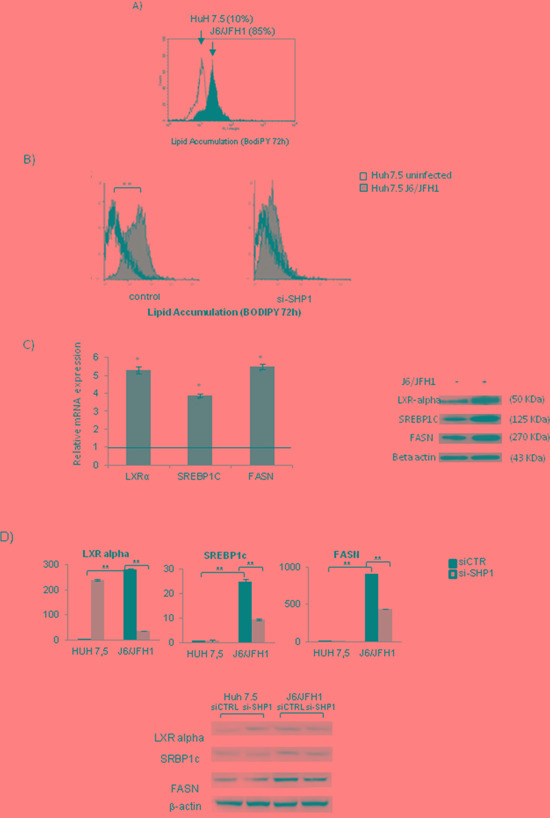
SHP1 and HCV related lipid accumulation **A.** FACS analysis of lipid accumulation (BODIPY) in J6/JFH1 HuH7.5 cells, 72 hours post infection. The results are representative of 3 independent experiments. **B.** FACS analysis of the effects of SHP1 silencing on cell lipid accumulation (BODIPY) (Huh7.5 J6/JFH1: 65 ± 5.82 vs siSHP1 Huh7.5 J6/JFH1: 3.65±1.32). The results are representative of 3 independent experiments. **C.** Real-time PCR and WB analyses of LXRα, SREBPc1 and FASN, 72 hrs post infection. The results, derived from 5 independent experiments, and are represented as mean ± S.D, *p<0.05. **D.** Real-time PCR and WB of LXRα, SREBPc1 and FASN, 72 hrs post-infection, in presence of si-SHP1. The results, derived from 5 independent experiments, are represented as mean ± S.D, **p<0.01.

Through FACS analysis we revealed a significant intracellular lipid accumulation in J6/JFH1 HuH7.5 when compared to uninfected Huh7.5 cells (86.56% ± 2,36 vs 10,16±1,25, p=0.032) (Figure 4A). After the treatment of J6/JFH1 Huh7.5 infected cells with si-SHP1, the lipid accumulation was significantly (**p<0.01) decreased, restoring the intracellular lipid content observed in the control cells (from 85%±8,56 in Huh 7.5 J6/JFH1 to 15%±2.36 in SHP1 siRNA, Figure 4B).

In Figure 4C we highlighted the increased mRNA and protein levels of the well-known steatogenic genes LXRα, SREBP1c and FASN, after 72h of infection. Treatment of infected cells with SHP1-siRNA determined a strong reduction (**p<0.01) of mRNA and protein levels relative to these genes (Figure 4D).

### SHP1 and EMT genes expression

To understand if SHP1 could be involved in HCV-related hepatic transformation, we looked at the expression levels of HNF4, E-cadherin and SNAIL, all implicated in the epithelial to mesenchymal transition (EMT).

**Figure 5:**
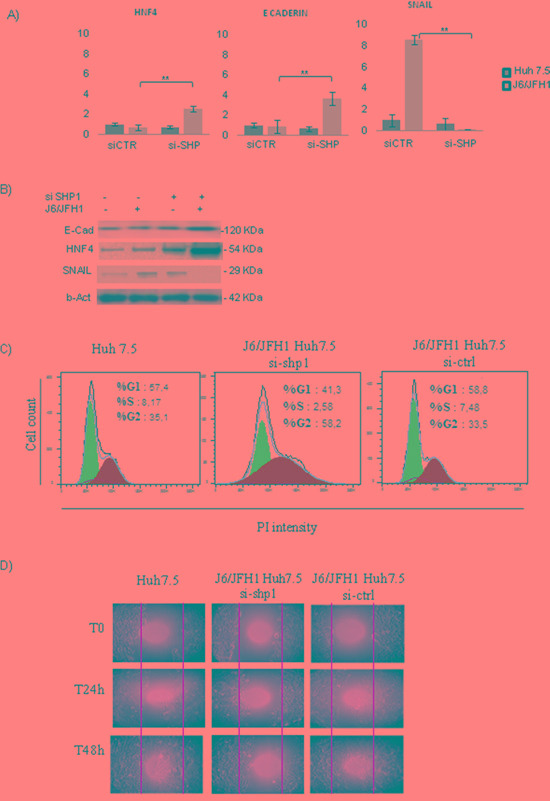
SHP1 and Epithelial Mesenchimal Transition (EMT) **A.** Real-time analysis of HNF4, E-Cadherin and SNAIL, in J6/JFH1 HuH7.5, before and after siSHP1. Data derived from three independent experiments, and are expressed as mean values± S.D, **p<0.01. **B.** WB analysis of HNF4, E-Cadherin and SNAIL, representative of 3 independent experiments. **C.** FACS analysis of J6/JFH1 HuH7.5 cell cycle, after 48hrs of siSHP1, using propidium iodide (PI) staining. **D.** *In vitro* scratch assay, after siSHP1. Representative microphotographs were taken after 24h and 48h from the initial scratch.

RT-PCR and proteins analysis in Huh7.5 and JFH1/J6 HuH7.5 cells, after 72hrs of HCV infection, didn’t reveal any regulation of the HNF4 and E-cadherin; on the other hand, a significant increase of SNAIL was reported (p<0.001). Interestingly, after SHP1 silencing, HNF4 and E-Cadherin expression significantly increase in HCV positive cells (**p<0.01), while SNAIL mRNA and corresponding protein decrease (**p<0.01) (Figure 5A-5B).

We performed also FACS analysis of Huh7.5 and JFH1/J6 HuH7.5 cell cycle, in the presence of si-SHP1. si-SHP1 treatment reduced the S-phase and significantly increased the G2/M phase; the G0G1 phase was significantly decreased, indicating that the cells had lower rates of growth and tended to be arrested at the G2/M transition (Figure 5C).

Finally, we performed an *in vitro* scratch assay, for 24 and 48hrs, on Huh7.5 or JFH1/J6 Huh 7.5, in the presence of si-Shp1. The microphotographs of the scratch width, taken at 24 and 48 hours after the initial scratch, showed that si-SHP1 is able to counteract the cells migration (Figure 5D).

## DISCUSSION

HCV is a major health problem since in the world almost 170 million individuals are chronically infected. Liver steatosis and oxidative stress play an important role in liver damage during HCV infection; accordingly, several transcriptional regulators of cholesterol and fatty acid metabolism have been shown to be implicated in HCV infection [22–23]. In a recent work, we investigated the expression profiles of genes associated to HCV-related steatogenesis in liver biopsies and PBMCs of HCV positive patients [19]. We highlighted the involvement of different Nuclear Receptor in this phenomenon, such as FXR, LXRs, HNF4. Interestingly, dysfunction of nuclear receptors signaling leads to a wide spectrum of proliferative, reproductive, and metabolic diseases, including obesity, diabetes, and cancers [24].

Because of their important physiologic roles in cancers, nuclear receptors (NRs) are emerging as targets for molecular diagnostic tests and cancer therapeutics. In particular, SHP1 cooperates in the regulation of NRs and transcription factors and plays a major role in the patho-physiology of metabolic disorders in the liver: SHP1 repressive activity is crucial in controlling the development of metabolic syndrome and fibrosis [25, 26]. Besides its metabolic regulatory function, it has been demonstrated that SHP1 has a tumor-suppressive activity, by inhibiting cellular growth and activating apoptosis, thus playing a critical role in the development of cancer [27].

In this study, we highlighted a significant up-regulation of SHP1 in chronic HCV positive patients, as well as in two different *in vitro* HCV infection systems. In our opinion, the most important result obtained by us was the proof of the direct interaction between SHP1 and the viral protein NS5A. Moreover, we performed molecular docking study, highlighting that the D1 domain of NS5A is that involved in the interplay between the two proteins of our interest.

Moreover, we highlighted the SHP1-dependent up-regulation of LXR, SREBP1c and FAS, and the related lipid accumulation. We also demonstrated a SHP1-dependent deregulation of some important markers of Epithelial to Mesenchymal Transition (EMT), such as HNF4, E-Cadherin, and SNAIL occurs, highlighting an important role of SHP1 in sensitizing liver cells to transformation.

Our results, apparently, seem in contrast with the known tumor suppressive effect of SHP1.

Conversely, we believe that, in light of our results, in the presence of HCV, SHP1 functions are altered, and partially lost. Based on our experiments, we believe that, in the presence of HCV, the typical functions of SHP1 change its course, being affected by the interaction with the NS5A viral protein, thus favoring HCV-related liver steatosis and transformation.

**Figure 6:**
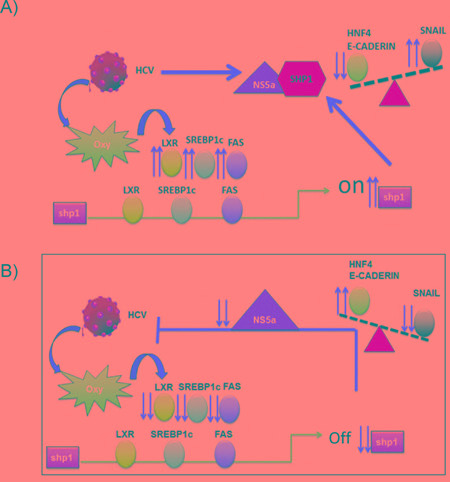
Schematic representation of the possible role of SHP1 in HCV-related liver transformation **A.** The up-regulation of LXRα, SREBP1c and FASN by HCV infection induces lipid accumulation, increasing SHP1 expression that, in turn interacts with NS5A, causing deregulation on the expression of EMT related proteins: HNF4-E, Cadherin and SNAIL. **B.** SHP1 silencing antagonizes lipid accumulation and the induction of EMT, restoring the basal levels of HNF4, E-Cadherin and SNAIL, in HCV infected cells.

One hypothesis is that HCV drives the up-regulation of LXRα, SREBP1c and FASN, causing lipid accumulation, increasing the expression of SHP1 that, in turn, interacts with NS5A viral protein, causing the deregulation on the expression of some EMT related proteins: HNF4, E-Cadherin and SNAIL (Figure 6). Accordingly, in HCV infected cells, the silencing of SHP1 reverted all the effects caused by HCV infection, down-regulating the pro-steatogenic genes, thus impairing lipid accumulation, and counteracting not only the up-regulation of the genes associated to EMT but also decreasing the cell migration of infected cells, as well as their S phase of the cell cycle and increasing the G2/M, thus hindering tumorigenesis.

Our data lead us to suggest that, during chronic active HCV hepatitis, SHP1 could be an effective target to hamper the progression of liver pathology towards HCC. To improve the knowledge on the fundamental mechanisms used by SHP1 to deregulate liver cells pushing the onset of HCV-related liver cancer, may give novel preventive or therapeutic strategy to counteract HCV-related HCC.

## MATERIALS AND METHODS

### Cell culture, constructs and reagents

The human hepatoma-derived cell line Huh7.5 were grown in Dulbecco's modified Eagle's medium (DMEM) supplemented with 10% fetal bovine serum, 100 U/ml penicillin and 100 mg/ml streptomycin and 2 mM L-glutamine (Lonza). J6/JFH1 cDNA (kindly provided by C. Rice, Rockefeller University) was used to generate the HCV cell culture model. As control of infection we used JFH-A4 (REP): Huh7.5 cells replicating the JFH-1 subgenomic replicon together with the luciferase reporter gene were a kind gift from R. De Francesco (INGM, Milan). G418 (0.8 mg/ml, Sigma Aldrich) was used to select JFH-A4 (REP)+ Huh7.5 cells. JFH1 A4 replicon (genotype 2) is an efficient cell culture system, composed of two variants of the HCV-IRES [nucleotides (nt) 1 to 377 or 1 to 389], the neomycin phosphotransferase (neo) gene, the IRES of the encephalomyocarditis virus, which directs translation of HCV sequences from NS2 or NS3 up to NS5B, and the 3 NTR. The cells expressing subgenomic replicon system are capable of mimicking the effect of wt virus without forming viral particles [28].

Antibodies against HCV NS5A (Austral Biologicals), Core (Affinity bioreagents, Pierce), human LXR alpha, SREBP1c, FAS, SHP1, HNF4 (Santa Cruz), E Cadherin, TWIST and SNAIL (Cell Signaling) and Beta-actin (Santa Cruz) were used.

### Patients

40 HCV infected patients and 20 healthy donors (HDs) were enrolled for these investigations, as reported in a previous article published by us (19). In particular, healthy liver tissues were obtained from patients that undergo abdominal surgery (e.s. cholecystectomy, appendectomy) and that don’t display any sign of liver inflammation. In this regard, we cooperate with the staff coordinated by prof. Luigi Masoni at the San Pietro Hospital, in Rome.

### Replication and infection assays

The chimeric J6/JFH1 virus was generated and used according to Lindenbach BD and collegues [29, 30]. Huh7.5 cells were infected (MOI=0.1) 1 day after seeding. After 3 days cells were harvested and processed for RNA (Trizol reagent, Thermo Scientific) and protein extractions.

### Real time PCR

cDNAs synthesis were prepared according to the manufacturer's instructions (thermo scientific) and Real time PCR reactions were performed with the 7500 Fast Real-Time (Applied Biosystems), using SYBR Green (Applied Biosystems) detection. β- actin was used as an internal control. The specific primers used for qPCR reactions, are listed in Table 1.

**Table 1:** qPCR primers

Gene bank and symbol		Sequences
NM_021969 SHP1	For	CCCAGCATACTCAAGAAGA
	Rev	GACTCCAGACAGCATTGA
JN180452.1 NS5A	For	AGA AAA TGG CCC TTT ACG ATG TG
	Rev	CAG GAA CTC GAC CCG CTG T
JN180452.1 HCV IRES	For	GCGAAAGGCCTTGTGGTACT
	Rev	CACGGTCTACGAGACCTCCC
NM_005693 LXRα	For	TGCCGAGTTTGCCTTGCTCATT
	Rev	GGAGGCTCACCAGTTTCATTAGCA
NM_004104 FASN	For	GCCGAGTACAATGTCAACAACCTG
	Rev	AGGTTGTCCCTGTGATCCTTCTTC
NM_004176 SREBP1c	For	CCATCGACTACATTCGCTTTCTGC
	Rev	CCGACACCAGATCCTTCAGAGATT
Z49825 HNF4	For	GAGAATGTGCAGGTGTTGACGATG
	Rev	CAGATATGCTCCAGTGATGTCGGA
NM_005983 SNAIL	For	TGTCAGATGAGGACAGTGGGAAAG
	Rev	GCCTCCAAGGAAGAGACTGAAGTA
NM_001017992 Beta-actin	For	GCACTCTTCCAGCCTTCC
	Rev	AGGTCTTTGCGGATGTCCAC
NM_004360.3 E Cadherin	For	GACACCCGATTCAAAGTG
	Rev	GGCGTAGACCAAGAAATG
NM_000474.3 TWIST	For	CTCAGCTACGCCTTCTCG
	Rev	ACTGTCCATTTTCTCCTTCTCTG

### SHP1 RNA interference

siRNA experiments were performed using small interfering RNA (siRNA) oligonucleotides against SHP1, using ON-TARGET plus (cod. LU-003410-00-0005) and ON-TARGET plus non-targeting as control (cod. D-001810-10-05) using DharmaFECT 4 Transfection Reagent (cod. T-2004-03) (Dharmacon, Inc., Lafayette, CO, USA) according to the manufacturer instructions.

To optimize and achieve the best results in siRNA transfection, we transfected JFH1 A4 replicon system and HuH7.5 J6/JFH1 cells with four different siRNA-SHP1 oligonucleotides (named A1-A4), finding that the siRNA A4 was more efficient to silence SHP1 without affecting cell viability (Figure S1). Thus, all described experiments were performed with si-RNA A4 oligonucleotide.

RNAs and proteins were harvested after 24 hours from transfection and processed for RNA (Trizol reagent, Thermo Scientific) and protein extractions.

### Flow cytometry analysis (FACS)

To analyze lipid accumulation after infection, cells were suspended and incubated with fluorescent probe BODIPY® (Thermo Scientific) at 4 °C for 30 min, 72h post infection and after SHP1 interference. The intracellular NS5A protein was analyzed in cells pre-incubated with FITC-conjugated anti-human NS5A or irrelevant control antibody for 30 min and washed twice in PBS. Flow cytometry analysis was performed by a FACScan and Cell QuestTM software.

After 24 hrs, the cell cycle of Huh7.5 cells and HuH7.5 JA4 Rep were performed in presence of si-SHP1 or si-Ctrl. The cells were washed with PBS and fixed in ice-cold ethanol (70% v/v). Then the cells were washed with PBS and permeabilized with Triton X-100 0.25% for 15 min. After washed with PBS, the cells were re-suspended in propidium iodide stain (PI, 10μg/mL Sigma-Aldrich) with RNase A (20μg/mL, Invitrogen). The cell suspension was added to a FACS tube and incubated at room temperature in dark for 1 hours. The DNA content was measured using an LSR Fortessa flow cytometer (Becton Dickinson) and analyzed FlowJo software (Treestar, Ashland, OR).

### Apoptosis assays

Apoptosis was evaluated by flow cytometry analysis of isolated nuclei, using a method that distinguishes nuclei from apoptotic, necrotic or viable cells, as previously described [31].

### Western blot analysis

For protein extraction, cells were suspended in RIPA lyses buffer plus protease and phosphatase inhibitors (Sigma-Aldrich). The protein extract was analyzed through SDS–PAGE and probed with different primary antibodies and horseradish peroxidase–conjugate secondary antibody (Jackson ImmunoResearch Laboratories, West Grove, PA) and detected by ECL plus (GE Healthcare).

### Immunoprecipitation assay

Cells were suspended in lyses buffer (10 mM Tris-HCl, pH 8.0, 0.5% NP40,) plus protease and phosphatase inhibitors (Sigma-Aldrich). 1 mg of lysates were incubated with 1 μg of SHP1 or NS5A antibodies at 4°C for 4 h and followed by 2 h of incubation with 30 μl protein G–Agarose beads (Roche). The beads were collected by centrifugation and washed four times with lyses buffer. A 2x SDS-PAGE sample buffer was added and samples were boiled at 95°C for 10 min. Thus, WB analyses were performed.

### Confocal analysis

Cells were grown on coverslips and fixed. Then were incubated with primary antibodies against NS5A and SHP1 for 1 h at room temperature and after with Alexa Fluor-conjugated secondary antibodies (Thermo Scientific) for signal detection. Coverslips were mounted in Slow-Fade Antifade (Thermo Scientific) and examined under a confocal microscope (Leica TCS SP2). Digital images were acquired by Leica software, and processed with ImageJ (National Institutes of Health). Co-localization analysis was performed by Image J software.

### *In vitro* scratch assay

Huh7.5 cells and HuH7.5 JA4 Rep in presence of si-SHP1 or si-Ctrl were plated onto a 6-well plate with DMEM media containing 10%-FBS and allowed to grow to ~90% confluence (24 hours). A 10μL pipette tip was used to make a scratch through the middle of the plate. Cells were gently washed (twice) with PBS and fresh media was added. The cells were allowed to migrate and images of the scratch width were taken at 24 and 48 hours after the initial scratch.

### Statistical analysis

Statistical analysis of data was performed using the SPSS statistical software system (17.0 Windows). The independent samples t-test analysis was performed; for non-parametric correlation, Spearman's rho correlation coefficient was calculated with a Bonferroni's correction. *p* values *p<0.05, **p<0.01, ***p<0.001 were considered statistically significant.

## SUPPLEMENTARY MATERIALS FIGURES AND TABLE


