# Supplementary material for: Small heterodimer partner 1 directly interacts with NS5A viral protein and has a key role in HCV related liver cell transformation
Source: Oncotarget. 2016 Sep 20;7(51):84575–86. doi: 10.18632/oncotarget.12144 (PMC5356682; doi:10.18632/oncotarget.12144)
Supplement: Supplementary file 1 [file oncotarget-07-84575-s001.pdf]

Small heterodimer partner 1 directly interacts with NS5A viral protein and has a key role in HCV related liver cell transformation

SUPPLEMENTARY FIGURES AND TABLE

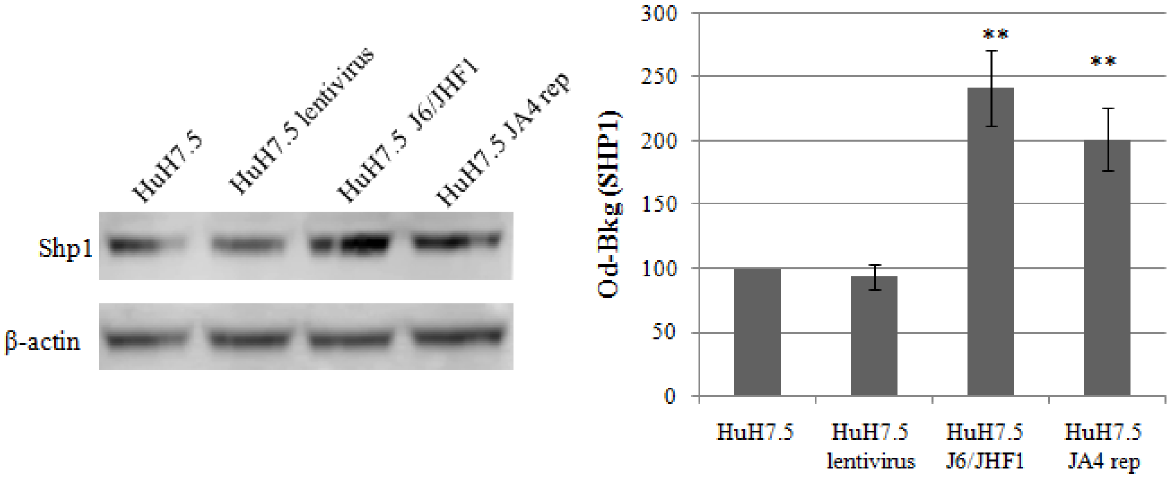

Supplementary Figure S1: Western blot and relative densiometric analysis of SHP1 expression in HUH7.5 transfected with a lentiviral empty vector, J6/JFH1and JFH1 A4 replicon system. Data are expressed as mean values ± S.D, **p<0.01. Beta-actin is used as a loading control.

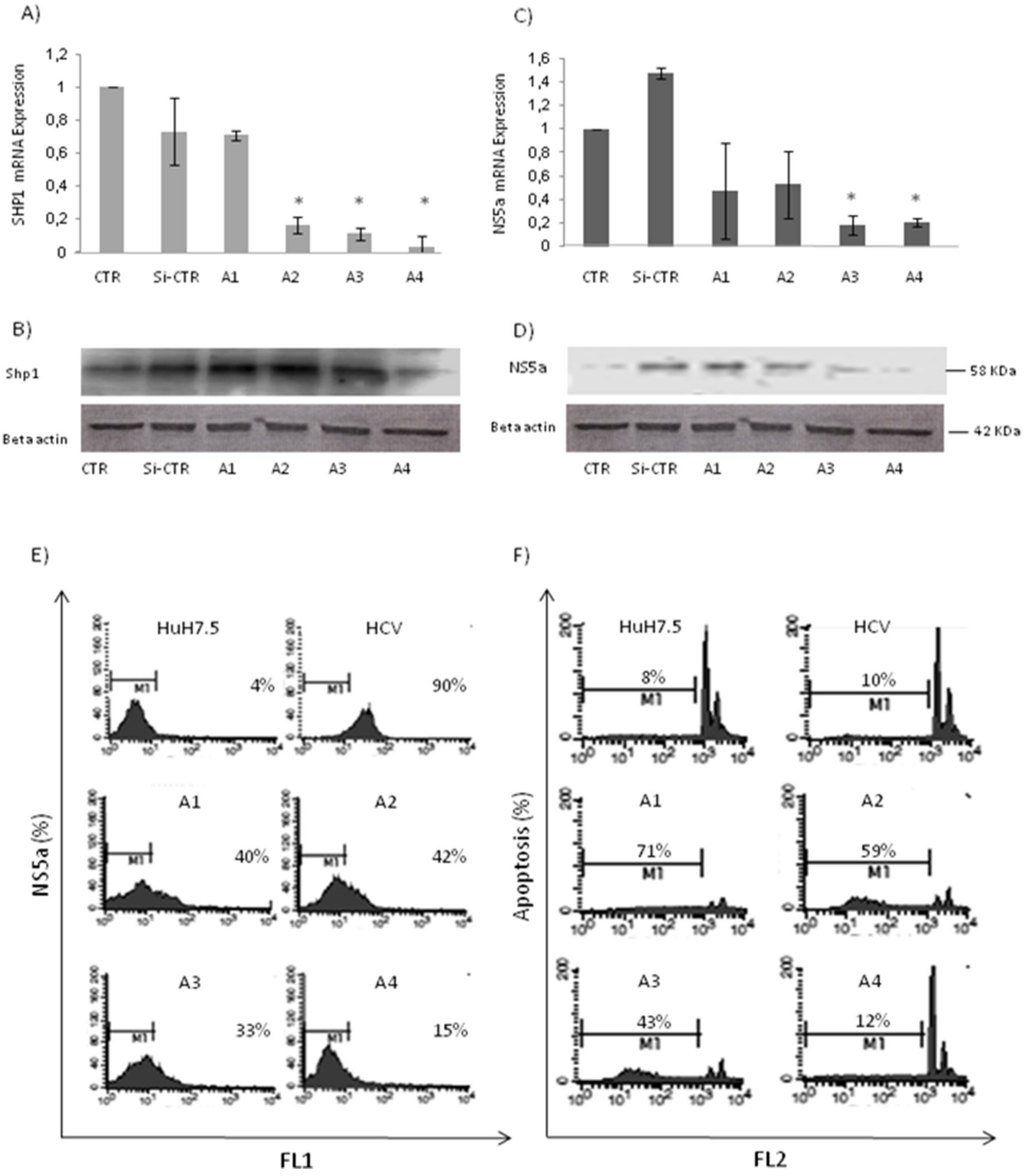

Supplementary Figure S2: SHP1 interfering efficacy in both HUH7.5 J6/JFH1and JFH1 A4 replicon system, testing four different siRNA-SHP1 oligonucleotides (named A1-A4). A-D. mRNA expression levels and Western Blot assay of SHP1 and NS5A. Data are expressed as mean values ± S.D, $^{**}p<0.01$. Beta-actin is used as a loading control. Immunoblot is representative of three independent experiments. **E-F.** FACS analysis of NS5A and apoptosis. The results are representative of 3 independent experiments.

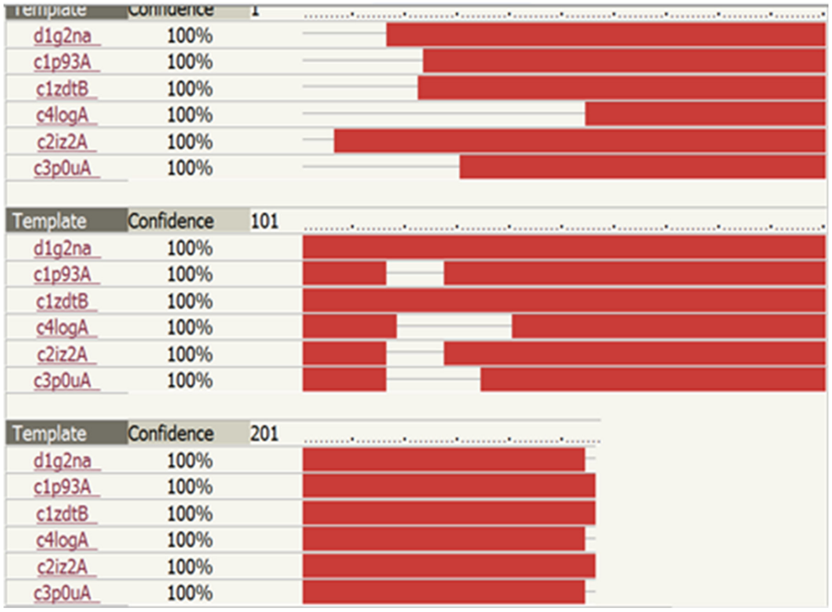

Supplementary Figure S3: We reported the six templates by their PDB codes used by Phyre server to model the SHP1 structure, the confidence percentages evaluated between SHP1 and template structures and the related alignment coverage indicating where the sequence was covered by each template, red-coded by the confidence of the match to that template overall.

Supplementary Table S1: We reported in bold the residues in SHP1 and in NS5A that are at the interface of the complex, and D1 in italic

SHP1
MSTSQPGACPCQGAASRPAILYALLS**SSLKAV**P**R**P**R**SR**C**LCRQHRPVQLCAP**H**RTCREAL
DVL**AK**T**VA**FL**RN**LPSFWQLPPQDQ**R**RLLQGCWGPLFLLGLAQDAVTFEVAEA**P**V**P**SILKK
ILLEEPSSSGGSGQLPDRPQPSLAAVQWLQCCLESFWSLELSPKEYACLKGTILFNPDVP
GLQAASHIGHLQQEAHWVLCEVLEPWCPAAQGRLTRVLLTASTLKSIPTSLL**GD**LF**FR**P**I**
IGDVD**IAGL**LG**D**MLLLR

***D1*-D2-D3** domains in **NS5A**
*LPGVPFFSCQRGYKG*___V___*WRGDGIMVTTCPCGAQITGHV*___KNGS___*MR*___IVG___*PKTCSN*___MWHGT___*FPINAHTTG*
*PCTPSPAP*___N___*YSRALWRVAAEEYVEVTRVGDFHYVTGMTTDNIKCPCQVPAPEFFTEVDGVRLHRYA*
*PACKPLLREEVTFQVGLNQYLVGSQLP*CEPEPDVTVLTSMLTDPSHITAETAKRRLDRGSPPSLAS
SSASQLSAPS**LKATC**TTRHDSPDADLIEANLLWRQEMGGNITRVESENKVVILDSFDPLRAEEDGG
EVSVPAEILRKPRKFPPALPIWARPDYNPPLLESWKDPDYTPPVVHGCPLPPAKAPPIPPPRRKRT
VVLTESNVSSALAELATKTFSGSGSSAVDSGTASAPPDQPS**DD**GDV
